# Factors affecting prognosis of status epilepticus among patients presenting to a tertiary care hospital

**DOI:** 10.12669/pjms.38.1.5195

**Published:** 2022

**Authors:** Wajid Jawaid, Muhammad Irfan, Sundus Mehtab Shafee, Sidra Javed Barry, Sayed Mustafa Mahmood Shah, Naila Shahbaz

**Affiliations:** 1 Wajid Jawaid, FCPS Neurology, Department of Neurology, Dow University of Health Sciences & Dr. Ruth K. M Pfau Civil Hospital, Karachi, Pakistan; 2 Muhammad Irfan, FCPS Neurology, Department of Neurology, Dow University of Health Sciences & Dr. Ruth K. M Pfau Civil Hospital, Karachi, Pakistan; 3 Sundus Mehtab Shafee, MBBS. Department of Neurology, Dow University of Health Sciences & Dr. Ruth K. M Pfau Civil Hospital, Karachi, Pakistan; 4 Sidra Javed Barry, MBBS. Department of Neurology, Dow University of Health Sciences & Dr. Ruth K. M Pfau Civil Hospital, Karachi, Pakistan; 5 Sayed Mustafa Mahmood Shah, MBBS. Department of Neurology, Dow University of Health Sciences & Dr. Ruth K. M Pfau Civil Hospital, Karachi, Pakistan; 6 Naila Shahbaz, FCPS Neurology, Department of Neurology, Dow University of Health Sciences & Dr. Ruth K. M Pfau Civil Hospital, Karachi, Pakistan

**Keywords:** Status epilepticus, Prognostic factors, Mortality, Morbidity, Refractory, Super-refractory

## Abstract

**Objectives:**

This study aimed to evaluate the etiology, outcomes and prognostic factors associated with status epilepticus (SE) admissions in Neurology Department of a tertiary care hospital.

**Methods:**

A retrospective review was performed on all SE admissions at Dr. Ruth K.M. Pfau Civil Hospital Karachi over a five-year period from July 2015 to June 2020. Demographic, clinical, and etiological factors were investigated for prognostic value. Statistical tests were applied to determine significant prognostic factors. A five percent significance level was used.

**Results:**

A total of 176 patients were included in the study. Mortality was reported in 22 cases (12.5%) and morbidity at six months was observed in 44 cases (25.0%). Male gender, previous history of SE, prolonged seizure duration, and late presentation to hospital were significantly associated with mortality (p<0.05). De novo cases of SE tended to be older (p=0.048) and were associated with morbidity at follow-up (p=0.000). The most common causes of epilepsy in our patients with SE were CNS infections (n=54) and Idiopathic epilepsy (n=34). Non-compliance to medicines/under-dosing was the most common provocative factor (n=68). Acute symptomatic causes of SE were more likely to be associated with greater morbidity (p=0.000). Refractory and super-refractory SE were strongly associated with higher mortality (p=0.000). A longer duration of hospital stay was associated with higher morbidity (p=0.000).

**Conclusion:**

Male gender, poor control of seizures, CNS infections, prolonged seizures, delayed hospital arrival and refractory/super-refractory status epilepticus were key determinants of mortality in our setting. Previous history of status epilepticus, and acute and symptomatic etiologies were associated with higher morbidity.

## INTRODUCTION

Status epilepticus (SE) is a neurological emergency characterized by continuous seizures for more than five minutes or recurrent seizure activity for more than five minutes without recovery between seizures.^1^ The estimated global incidence of SE ranges from 1.29 to 73.7/100,000 with wide variation across location and type of study.^2^ Within developing countries, the pooled crude annual incidence is reported as 13.8 per 100,000.^3^,^4^ Despite advances in understanding the clinical course of SE and optimal management, short-term mortality remains as high as 3-40% depending on the center and type of SE encountered.^5^

An approach to further delineate components of SE with a bearing on clinical course and management has been made by the International League Against Epilepsy (ILAE) by distinguishing separate axes for SE on the basis of semiology, EEG correlates, etiology, and age at onset.^6^ This has fueled scope for further study into the broad array of prognostic factors that may aid in identifying SE patients considered high-risk and guide optimal treatment decisions. Matching the degree of treatment and intensity of care to the severity of SE encountered will not only streamline delivery of care, but will avoid the risks carried by overtreatment of patients with milder disease.^7^

There is a paucity of data relaying the epidemiology, prognosis and outcomes of SE presenting within South-Asia. This is particularly true of Pakistan where only a couple of pediatric studies on SE have been published.^8^,^9^ This has significant implications for the development of a sound clinical approach given the unique demands of the region. We aimed to fill this gap by conducting a retrospective observational study of SE admissions over a five-year period to delineate the demographic, etiological, and clinical characteristics of SE patients presenting to a tertiary care hospital in Karachi, Pakistan. The outcomes of interest included in-hospital mortality and 6-month morbidity, for which key prognostic variables were investigated.

## METHODS

### Study design and duration:

A retrospective observational study was conducted at the Department of Neurology, Dr. Ruth K.M. Pfau Civil Hospital Karachi. All patients admitted in Neurology ICU with status epilepticus for a five-year period from July 2015 to June 2020 were included.

### Patient Management:

Patients were managed under a standardized treatment protocol in keeping with current treatment algorithms, which included initial administration of IV benzodiazepine (diazepam/midazolam), followed by stepwise administration of IV anti-seizure drug (phenytoin/valproic acid/levetiracetam) and IV anesthetic agent (midazolam infusion/propofol infusion) as needed. Administered doses of these drugs were variable, based on the patients’ weight and response to the drug. Airway was secured; continuous monitoring was instituted for all patients.

### Data collection:

The department’s record was used to collect data on demographic variables such as age, gender and residence of the patients. Clinical course, in-hospital management and response to therapy were also recorded from the data. Follow-up assessment of sequelae and patients’ progress over the following six months completed our data collection from the records.

Etiologies of SE were divided temporally as acute or chronic, and considered separately as cryptogenic (having no identifiable causative factor) or symptomatic (occurring due to another distinct disease entity).^10^ SE was classified by semiology as per the recent ILAE classification and guidelines.^6^

### Inclusion criteria:

Patients aged 12 years or more who were admitted with status epilepticus in the study setting during the study period were included.

### Exclusion criteria:

Patients who were initially managed in some other department or the ones who were taken out against medical advice before completion of treatment were excluded.

### Definitions:

Status epilepticus was defined as continuous seizure activity for at least five minutes or recurrent seizures for more than five minutes without recovery between seizures. Refractory status epilepticus was defined as SE episodes not controlled by first-line and second line AED, thus requiring IV anesthetics. SE remaining for 24 hours or more following anesthetic administration was defined as super-refractory SE.^11^ Morbidity in this study was considered when patients developed cognitive impairment, motor deficits or active epilepsy (alone or in combination) as a complication of SE. Scar epilepsy refers to delayed presentation of epilepsy as a sequelae of previous cerebral insult like tumor, trauma, surgery, stroke etc.

### Statistical Analysis:

Statistical Analysis was performed using IBM SPSS version 21. Descriptive statistics and frequencies were calculated. The normal distribution of the variables was tested by one sample Kolmogorov-Smirnov test. Chi-squared test with 95% confidence interval was used to compare categorical variables. Analysis of variable with small cell scores was computed using Fisher’s Exact Test or Likelihood Ratio, as appropriate. Non-parametric tests were used to compare differences in continuous variables across groups. A five percent significance level was used throughout the study.

### Ethical Considerations:

The study was approved by the Institutional Review Board of Dow University of Health Sciences on 15^th^ September, 2021 [IRB-2100/DUHS/Approval/2021/523].

## RESULTS

**Fig.1:**
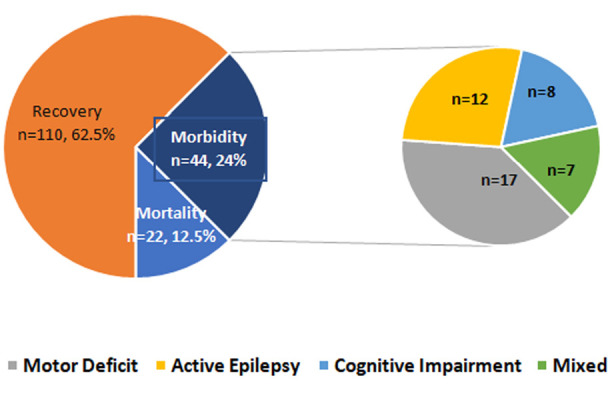
Prognosis among study population.

There was a total of 176 admitted patients with SE during the study period. Mortality was reported in 22 cases (12.5%) and morbidity was reported in 44 cases (25.0%). Further grouping of different morbid sequelae with their frequency is represented in Fig.1.

**Fig.2:**
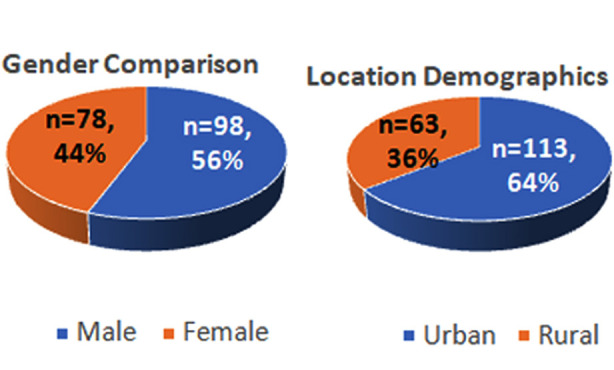
Socio-demographic characteristics of study population.

The median age of our study population was 24 (IQR=15.0-31.75).Gender and residential information is given in Fig.2. Male gender was strongly associated with mortality (p=0.002) but not morbidity (p=0.356). A majority had not received any formal education (60%). A previous history of status epileptic attacks was significantly associated with mortality (p=0.004). Other socio-demographic variables were not significantly associated with mortality or morbidity (p>0.05) (Table-I).

**Table I:** Socio-demographic characteristics of study population.

Characteristics	Frequency n (%)	Association with Morbidity (p-Value)	Association with Mortality (p-Value)
Males	98 (55.7)	0.356	0.002
Uneducated	105 (59.6)	0.110	0.791
Primary education	41 (23.2)		
Secondary education	28 (16.0)		
Graduated	2 (1.1)		
Family history of epilepsy	9 (5.1)	0.402	0.116
History of status epilepticus	24 (13.8)	0.808	0.004

**Fig.3:**
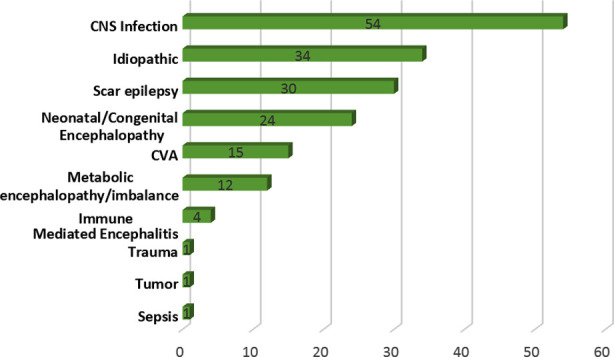
Etiology of epilepsy in study population.

The most common causes of epilepsy in our patients with SE were found to be CNS infections (n=54) and Idiopathic epilepsy (n=34) (Fig.3). The etiological characteristics of SE are outlined in Table-II. Most cases of SE occurred after a previous history of epilepsy (n=140,79.5%). De novo cases of SE were associated with greater morbidity (p=0.00), but not with greater mortality (p=0.862). Older age was significantly associated with a presentation of de novo SE (0.048). Symptomatic causes of SE were associated with greater morbidity as compared to cryptogenic SE (p=0.009); no such association was found with respect to mortality (p=0.171). Acute causes of SE were similarly associated with higher likelihood of morbidity (p=0.00), but not with higher mortality (p=0.137). A longer duration of hospital stay was associated with the occurrence of SE due to an acute etiology (p=0.000) and symptomatic SE (0.003).

**Table II:** Etiological characteristics of study population.

Characteristics		(n)	(%)	Association with Morbidity(p-Value)	Association with Mortality(p-Value)
History of prior seizures	De novo status epilepticus	36	20.4	0.00	0.862
	History of epilepsy	140	79.5	>0.05	>0.05
Type of etiology	Cryptogenic	38	21.5	>0.05	0.108
	Symptomatic	138	78.4	0.009	0.171
Time course of etiology	Acute	56	31.8	0.00	0.137
	Chronic	120	68.1	>0.05	>0.05

Cases of SE which were precipitated following a defined provocative factor are summarized in Table-III. Non-compliance to medicines/under-dosing was the most common provocative factor (n=68, 38.6%), followed by infective etiologies (n=55, 31.3%). Highest mortality and morbidity rate were seen in infective etiology(54.5% and 27.2% respectively (Table-III).

**Table III:** Provocative factors associated with onset of status epilepticus.

Provocative Factor	Frequency n (%)(n=176)	Morbidity n (%)(n =44)	Mortality n (%)(n=22)
Noncompliant to Meds/ Under-dosed	68 (38.6)	8 (18.2)	5( 22.7)
Infective etiology	55 (31.3)	12 (27.2)	12 (54.5)
Unprovoked	20 (11.3)	8(18.8)	1 (4.5)
Vascular event	15 (8.5)	9 (20.4)	1 (4.5)
Metabolic derangement	12 (6.8)	5 (11.3)	2 (9.0)
Immune-mediated	4 (2.2)	1 (2.2)	0 (0.0)
Trauma	1 (0.6)	0 (0.0)	1 (4.5)
Tumor	1 (0.6)	1 (2.2)	0 (0.0)

**Fig.4:**
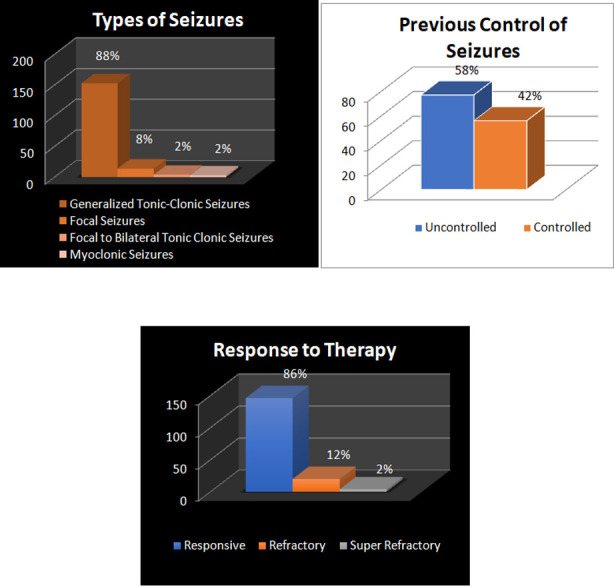
Clinical characteristics of seizures in SE patients.

The clinical characteristics of SE patients are illustrated in Fig-4.. SE with prominent motor phenomenon (generalized tonic clonic seizures) was the most common type (87.9%). Most patients presenting with SE reported poor seizure control in the preceding three months (58%), which was associated with higher morbidity at follow-up (p=0.000). Most cases of SE were found to be responsive to medical management (85.9%), while the remaining were categorized as having refractory or super-refractory status epilepticus. Type of seizure in SE was not significantly associated with mortality nor morbidity (p>0.05). Refractory and Super-refractory SE status was strongly associated with higher mortality (p=0.000).

The temporal profile of seizures are illustrated in Table-IV. The median hospital stay was seven days (IQR=4.25-10.75). Patients with a prolonged seizure duration and longer interval to hospital presentation were associated with higher mortality (p=0.000) and (p=0.000) respectively.

**Table IV:** Temporal Profile of Seizures.

Characteristics	Median (IQR)	Association with Morbidity (p-Value)	Association with Mortality (p-Value)
Seizure Duration	60 minutes (30-65)	0.842	0.00
Hospital Stay	7 days (4.25-10.75)	0.00	0.115
Time to Hospital Arrival	45 minutes (30-120)	0.712	0.001

## DISCUSSION

Status epilepticus is the second most common neurological emergency.^12^ “Time is brain” is not only a cliché when it comes to this potentially devastating condition that has bearing on the patients and their families.^13^ When dealing with an adversary, it is of utmost importance to know the arsenal at their disposal. This is where this study plays its role by outlining not only the prognostic factors in SE, but also the clinical, etiological, and demographic profile of SE patients presenting in a tertiary care hospital in Karachi over a five-year period.

The case fatality rate observed in this retrospective cohort was 12.5%. This is in line with the case fatality rate in developing countries of 15.6% as reported by a meta-analysis on the subject.^3^ Our study reported many potential factors implicated in the prognosis of SE and the significance of the causative etiology in SE outcomes. Male gender was found to be significantly associated with higher mortality (25.6% vs 19.4% in females). Multiple studies have documented a male preponderance in the occurrence of SE.^14^,^15^ Yet the effect of gender on outcomes remains unclear in the literature, with some studies reporting no effect^5^,^7^,^16^, higher fatality rate in females,^4^ or greater mortality in males.^17^ It is clear that acute symptomatic etiologies are associated with worse outcomes in SE patients, and the higher incidence of such etiologies in men may explain the higher incidence in the literature,^18^ and the poorer outcome in this study.

Advancing age trended towards poorer functional outcomes in our study. The relationship of advancing age and poor outcomes in SE has been clearly documented, which has led to the incorporation of age into various prognostic scoring systems for SE including the status epilepticus severity score (STESS) and the Epidemiology-based Mortality Score in Status Epilepticus (EMSE).^19^,^20^

A longer duration of SE and longer interval to hospital presentation were found to be significantly associated with greater mortality in this study. In a recent review Alkhachroum et al., discussed multiple studies documenting the same. They emphasized that the effect of SE duration on outcomes was strongly influenced by the underlying etiology.^18^ Our hospital serves as a major tertiary-care referral center at a city and provincial level, therefore more than one-third of SE presentations we received were from rural centers. This throws light on lack of facilities in the rural areas necessitating referral to our hospital,^21^ thereby increasing the time to receive appropriate management due to distance and adversely affecting the outcomes. Further study into the prevalence of SE in rural settings and implications for timely care are therefore warranted.

De novo cases of SE and SE arising from a symptomatic disease process were positively associated with worse functional outcomes at six-month follow-up. De novo cases of SE were also associated with older age. These findings are consistent with those reported by Lui et al, from an 11-year retrospective study conducted in Hong Kong,^22^ and are likely attributable to the acute symptomatic etiologies which accompany de novo and symptomatic SE. Further analysis of the etiologies underlying presentations during our study period confirmed the same; CNS infection was more strongly associated with worse outcomes as compared to SE due to cryptogenic epilepsy or non-compliance to medicines (p<0.005). A recent meta-analysis has also shown CNS infections to be an important cause of SE in the developing world, likely due to unsanitary conditions and inconsistent provision of healthcare.^3^ This has significant implications for the creation of a regional action plan in developing countries where proposals to address the burden of SE must address comprehensive infection-control practices in the region. Scar epilepsy was the third-most common etiology of epilepsy among our SE patients. The cerebral insults resulting in scar epilepsy are often result of a co-morbid condition like diabetes, hypertension, obesity or lifestyle factors like smoking, helmet-less bike riding etc.

Refractoriness of SE episodes was associated with mortality, whereas a longer duration of hospital stay was associated with poorer functional outcomes. A review by Nelson et al, noted that while the most common causes of SE may vary across various settings, the etiology of refractory SE appears to be similar to that of non-refractory SE.^11^ Our experience and analysis are in agreement with these observations. In the same review it is noted that super refractory SE may have a different etiological profile and commonly arises in the setting of encephalitis.^11^ This is especially alarming as a recent study from our centre found CNS infections as among the most common causes of symptomatic epilepsy in both pediatric and adult age group patients in our set-up.^23^ Among the four cases of super refractory SE in this study, three were secondary to CNS infections. The need of the hour is to prevent super-refractory status epilepticus by controlling the risk factors in the population, as mortality rate in these cases ranges from 30 to 50%.^24^

### Limitations of the study:

This was a retrospective study confined to one tertiary care hospital. Moreover, our set-up lacks the resources for continuous EEG monitoring of patients under ICU care. Continuous EEG monitoring has been advocated as an effective means of early detection of epileptic activity in patients admitted in the ICU, particularly for non-convulsive seizures, and can allow for the prognostication of patients at the bedside.^25^

## CONCLUSION

Prognosis of patients presenting with status epilepticus is influenced by socio-demographic and clinical variables. Male gender, poor control of seizures, CNS infections, prolonged seizures, delayed hospital arrival and refractory/super-refractory status epilepticus were key determinants of mortality in our setting. Previous history of status epilepticus and cases with acute and symptomatic etiologies were associated with higher morbidity. Public awareness programs should be conducted to emphasize on modifiable risk factors including drug compliance and seizure control as these can alter outcome of patients. More local studies are required regarding prognostic factors to provide high sensitivity and specificity in predicting outcomes of status epilepticus.

### Authors’ Contribution:

**WJ:** Conception and design, data acquisition, drafting and critical revision of the manuscript; responsible and accountable for the accuracy and integrity of the work.

**MI:** Design, data acquisition, critical revision of the manuscript.

**SMS:** Design, data acquisition, drafting the manuscript.

**SJB:** Design, data acquisition, drafting the manuscript.

**SMMS:** Data analysis and interpretation, drafting the manuscript.

**NS:** Conception and design, critical revision of the manuscript.
